# Xanthoxyline prevents aging and neuronal damage by activating autophagy and DAF-16 expression in *Caenorhabditis elegans*

**DOI:** 10.1080/19768354.2025.2549756

**Published:** 2025-08-22

**Authors:** Hyemin Kim, Saebyeol Lee, Hak Kyun Kim, Sang-Kyu Park

**Affiliations:** a Department of Medical Sciences, General Graduate School, Soonchunhyang University, Asan, Republic of Korea; b Department of Life Science, Chung-Ang University, Seoul, Republic of Korea; c Department of Medical Biotechnology, Soonchunhyang University, Asan, Republic of Korea

**Keywords:** Xanthoxyline, aging, Parkinson’s disease, autophagy, DAF-16

## Abstract

Xanthoxyline, a plant-derived phytochemical, has anti-bacterial, anti-fungal, and anti-cancer activities. We intended to investigate the effect of xanthoxyline on the response to oxidative stress, aging, and Parkinson’s disease. The effects of dietary supplementation with xanthoxyline on stress response and aging were examined *in vivo* using *Caenorhabditis elegans* as a model system. Genetic analysis using mutants, RNAi, and quantitative RT–PCR was performed to identify underlying mechanism involved in xanthoxyline-induced longevity. Animal disease models were employed to examine the effect of xanthoxyline on Parkinson’s disease. Xanthoxyline increased resistance to the oxidative stress induced by H_2_O_2_. The mean lifespan of worms was significantly increased by supplementation with xanthoxyline. The lifespan-extending activity of xanthoxyline was not accompanied by reduced fertility. Xanthoxyline delayed the age-related decline in motility. Interestingly, the expression of two longevity-assuring genes, *hsp-16.2*, and *sod-3*, was increased by xanthoxyline supplementation. Genetic analysis suggested that lifespan extension by xanthoxyline was mediated by activation of autophagy and required DAF-16. In a model of Parkinson’s disease, degeneration of dopaminergic neurons was prevented by supplementation with xanthoxyline, in a manner dependent on DAF-16. Taken together, we concluded that xanthoxyline exerts an anti-aging activity, possibly by activating the DAF-16-dependent stress response, and reduces the risk of Parkinson’s disease, in a manner mediated by DAF-16. Xanthoxyline shows promise for the development of novel nutraceuticals against aging and Parkinson’s disease.

## 1. Introduction

Aging is one of the most complex biological processes and involves various biochemical, genetic, and pathophysiological changes. A number of theories have been suggested to explain this universal, progressive, and irreversible biological phenomenon. The genetic control theory claims that the rate of aging and lifespan are encoded in an individual’s genome, and that genomic instability is the major causal factor of aging (Fossel 1998). Patients with Werner syndrome, a progeria involving accelerated aging, exhibited various aberrations in their genomes, including chromosome breaks and fusions (Shamanna et al. 2017). The telomere theory emphasizes the role of telomeres, repetitive sequences at the end of each chromosome that shorten after every cell division (Fossel 1998). Genetic manipulation to maintain telomere length conferred longevity in model organisms (Morin 1997). In 1961, Hayflick reported that human cells can divide only approximately 50 times and suggested the Hayflick-limit theory to understand the relationship between cell cycle regulation and cellular senescence (Shay & Wright 2000). The endocrine theory focuses on internal hormones controlling the rate of aging. Insulin/IGF-1-like signaling is a key hormonal action regulating lifespan and age-related physiological alterations in *C. elegans* and is functionally conserved in other organisms (Tatar et al. 2003). The best-known theory of aging is the free radical theory developed by Harman in 1956 (Harman 1956). Cells produce free radicals, such as superoxide and hydrogen peroxide, as byproducts of essential metabolism. Those free radicals attack cellular macromolecules and cause oxidative damage to DNA, protein, and lipids. The free radical theory suggests that accumulated oxidative damage gives rise to dysfunction in cells and organs, eventually leading to death. However, no single theory can explain the aging process, and the aging theories are interconnected.

Based on the free radical theory of aging, research has focused on interventions to reduce cellular oxidative damage and so retard aging. Reactive oxygen species (ROS), byproducts of mitochondrial ATP generation, are free radicals that cause cellular oxidative damage. Antioxidant enzymes, such as catalase (CAT), superoxide dismutase (SOD), and glutathione peroxidase (Gpx), transform ROS to non-harmful chemicals. The effects of genetic manipulation of antioxidant enzymes have been investigated in model organisms. Knockout of CAT or SOD reduced resistance to oxidative stress and lifespan in *Drosophila melanogaster*, whereas transgenic animals with extra copies of antioxidant genes did not have increased lifespan (Orr & Sohal 2003). Simultaneous over-expression of CAT and SOD significantly prolonged lifespan of *Drosophila melanogaster*, albeit only in short-lived strains (Sohal et al. 1995). In mice, knockout or transgenes of antioxidant genes, including CAT, SOD, and Gpx, did not affect lifespan (Perez et al. 2009). By contrast, neuron-specific expression of human SOD in *Drosophila melanogaster* and mitochondria-targeted expression of human CAT in mice resulted in longevity phenotypes (Parkes et al. 1998; Schriner et al. 2005).

ROS can be scavenged using antioxidant compounds. Vitamin E supplementation increased lifespan in *C. elegans* and *Drosophila melanogaster*, but showed inconsistent results in mice (Ernst et al. 2013). Several phytochemicals, such as resveratrol, quercetin, and catechin, can extend the lifespan and modulate age-related physiological changes in *C. elegans* (Howitz et al. 2003; Wood et al. 2004; Pietsch et al. 2009; Saul et al. 2009). Supplementation with phlorizin or fisetin increased lifespan in a manner involving the DAF16-induced stress response and activated autophagy, and reduced the risk of age-related diseases (Park et al. 2022; Park & Park 2022). Dietary intake of phospholipids, such as phosphatidylcholine, phosphatidylserine, and phosphatidylethanolamine, reduced susceptibility to oxidative stress, delayed the age-related decline of motility, and increased lifespan by suppressing insulin/IGF-1-like signaling (Kim et al. 2019; Kim & Park 2020; Park et al. 2021). Ezetimibe, a cholesterol-lowering drug, exerted a protective effect in models of Alzheimer’s disease and diabetes mellitus and prolonged lifespan mimicking dietary restriction (DR) (Park et al. 2023). The effects of antioxidant compounds on lifespan are related to their chemical characteristics and vary.

Xanthoxyline (2'-hydroxy-4’,6'-dimethoxyacetophenone) is a phenolic phytochemical produced by some plants, including *Zanthoxylum piperitum* and *Sebastiana schottiana*. It inhibits plant growth and has anti-fungal, anti-spasmodic, and anti-tumor activities (Cechinel Filho et al. 1995; de Carvalho et al. 2018). Xanthoxyline has been reported to reduce tumor weight and to have anti-angiogenic activity (Elhady et al. 2021). A ruthenium-xanthoxyline complex showed potent cytotoxicity against liver and colon cancer cells (de Carvalho et al. 2018; Santos et al. 2023). Interleukin-6 and acetylcholinesterase, which are involved in the progression of Alzheimer’s disease, were inhibited by xanthoxyline (Kaur & Bansal 2022). In this study, we investigated the effect of xanthoxyline on the responses to oxidative, heat, and ultraviolet (UV) stressors and aging *in vivo*. Lifespan, fertility, and age-related decline of motility were compared between the untreated control and xanthoxyline-treated groups. Genetic mutants, knockdown, and expression analysis were employed to identify the mechanisms underlying the effect of xanthoxyline. We evaluated xanthoxyline as a novel nutraceutical for age-related diseases using genetic models of Parkinson’s disease (PD).

## 2. Materials and methods

### 2.1. Worm strains and culture conditions

*C. elegans* strains including wild-type N2 were purchased from the Caenorhabditis Genetics Center (CGC, Minneapolis, USA). CL2070 (*dvIs70* [*Phsp-6.2::GFP, rol-6*]) and CF1553 (*muIs84* [*Psod-3::GFP, rol-6*]) were used for measurement of *in vivo* HSP-16.2 and SOD-3 expression, respectively. Two long-lived mutants, *age-1* (*hx546*) and *eat-2* (*ad465*), have different lifespan-extending pathways. To assess autophagy, BC12921 (*sIs10729* [*rCes T12G3.1::GFP*, *pCeh361*]) was employed. BZ555 (*egIs1* [*dat-1p::GFP*]) worms, which express green fluorescence protein (GFP) in dopaminergic neurons, were used to assay dopaminergic neurodegeneration. Worms were grown at 20°C on nematode growth medium (NGM) (25 mM NaCl, 2.5 mg/mL peptone, 50 mM KPO_4_, 5 μg/mL cholesterol, 1 mM CaCl_2_, 1 mM MgSO_4_, and 1.7% agar). *Escherichia coli* OP50 was provided as the food source. Xanthoxyline (Sigma Aldrich, Cat. No. 630586, St. Louis, MO, USA) was dissolved in sterilized distilled water.

### 2.2. Responses to environmental stressors

To induce oxidative stress, 30 age-synchronized 3-d-old adult worms were transferred individually to wells of a 96-well plate containing 2 mM hydrogen peroxide (H_2_O_2_) in S-basal medium without cholesterol (5.85 g of sodium chloride, 1 g of potassium phosphate dibasic, and 6 g of potassium phosphate monobasic in 1 L of sterilized distilled water). After 8 h of incubation, survival was recorded. Heat and UV stresses were applied to age-synchronized 3-d-old worms (n = 60) by placing them at 35°C for 8 h and under UV (20 J/cm^2^/min) for 1 min, respectively. Live and dead worms were counted daily until all the worms were dead.

### 2.3. Lifespan assay

Three d after hatching, 60 randomly selected worms were transferred to fresh NGM plates containing 5-fluoro-2’-deoxyruridine (12.5 mg/L) to prevent internal hatching. The numbers of live and dead worms were counted daily. Lost, killed, or internally hatched worms were excluded from the analysis. Kaplan-Meier analysis was used for statistical analysis of lifespan.

### 2.4. Fertility assay

Twelve mother worms were randomly selected at 48 h after hatching. Each mother worm was transferred to a fresh NGM plate (1 worm/plate) and incubated at 20°C to lay eggs. After 24 h, mother worms were individually transferred to fresh NGM plates. Eggs laid on NGM plates were incubated at 20°C for 48 h, and progeny hatched from the eggs were counted under a microscope. This process was repeated throughout the gravid period.

### 2.5. Age-related decline of motility

Age-synchronized 500 worms (50 worms/plate) were prepared. The locomotive activity of 100 worms (2 plates) was monitored at 5 d after hatching, and classified into phase 1, active locomotive activity in the absence of any stimulus, phase 2, locomotive activity only in the presence of a mechanical stimulus, and phase 3, head-limited motility in the presence of a mechanical stimulus. We repeated that with another 100 worms with a 5-d interval until 25 d after hatching. The number of thrashing movements of the worms (n = 15) per min in M9 buffer was determined.

### 2.6. Expression of longevity-assuring genes

Age-synchronized 7-d-old CL2070 and CF1553 worms were anesthetized with 1 M sodium azide on glass slides coated with 2% agarose and examined under a fluorescence microscope. To quantify fluorescence, 20 randomly selected worms were transferred to a 96-well plate, and fluorescence intensity was measured using a fluorescence multi-reader (Infinite F200, Tecan, Grodig, Austria) (n = 20).

### 2.7. RNAi

RNAi clones for each gene were obtained from the Ahringer RNAi library (Kamath et al. 2003). Double-stranded RNA synthesis was induced by adding isopropyl-β-D-thiogalactoside (IPTG, Sigma-Aldrich) to bacterial cultures. Cultured RNAi clones were spread on NGM plates as a food source. An empty vector (EV) clone served as the negative control.

### 2.8. Quantitative RT–PCR

Toral RNA extraction and quantitative RT–PCR was performed as described previously with following modifications (Fu et al. 2024). Total RNAs were extracted from 9-d-old worms using TRIzol reagent (Thermo) and converted to cDNA. Quantitative PCR was performed using a ReverTraAce qPCR RT Master Mix (TOYOBO), 2X SyGreen Mix Hi-ROX (qPCRBIO), and a StepOnePlus Real-Time^TM^ PCR System (Applied Biosystems). Relative expression levels were calculated using the 2 ^– ΔΔCt^ method. The primer pairs, including *ama-1* for normalization, are shown in Supplementary Table 1.

### 2.9. Measurement of autophagic activity

Age-synchronized 3-d-old BC12921 worms (n = 20) expressing fluorescent SQST-1 were treated with xanthoxyline at 20°C for 48 h. The worms were placed on a glass slide coated with 2% agarose and immobilized with 1 M sodium azide. The worms were visualized by fluorescence microscopy and analyzed quantitatively using Image-J software.

### 2.10. Degeneration of dopaminergic neurons

Dopaminergic-specific neurodegeneration was induced using 50 mM 6-OHDA (6-hydroxydopamine) as reported previously (Park et al. 2022). A fluorescence microscope fitted with a 470/22 nm excitation filter and a 525/50 nm emission filter was used to observe the degeneration of dopaminergic neurons (n = 20). Image-J software was used to quantify fluorescence intensity.

## 3. Results

### 3.1. Xanthoxyline increased resistance to oxidative stress and extended lifespan

**Figure 1.**
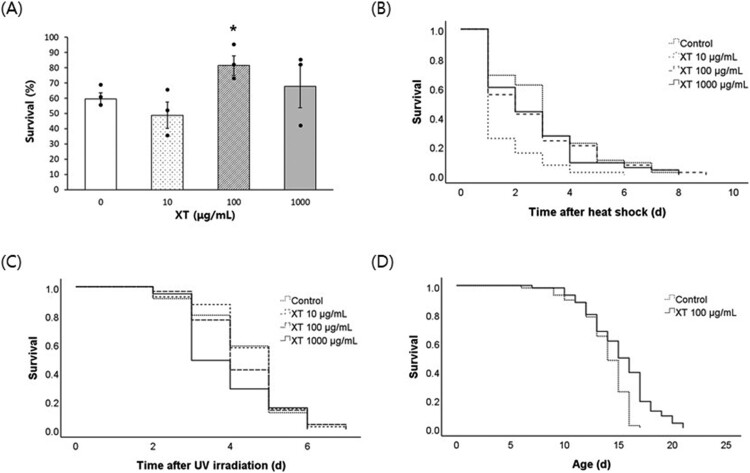
Effect of xanthoxyline on the responses to environmental stressors and lifespan. (A) Age-synchronized 3-d-old worms were treated with xanthoxyline for 24 h and their survival was monitored after 8 h of exposure to oxidative stress (2 mM H_2_O_2_). Survival of worms was compared between the untreated control and xanthoxyline-treated groups after 8 h of 35°C heat shock (B) or 1 h of UV irradiation (20 J/cm^2^/min) (C). (D) Lifespans of age-synchronized untreated control worms and worms treated with 100 μg/mL were compared. Error bars indicate standard errors. Kaplan-Meier analysis was used for statistical analysis of survival curve. XT, xanthoxyline; *, statistically significant compared to the control (*P* < 0.05).

**Figure 2.**
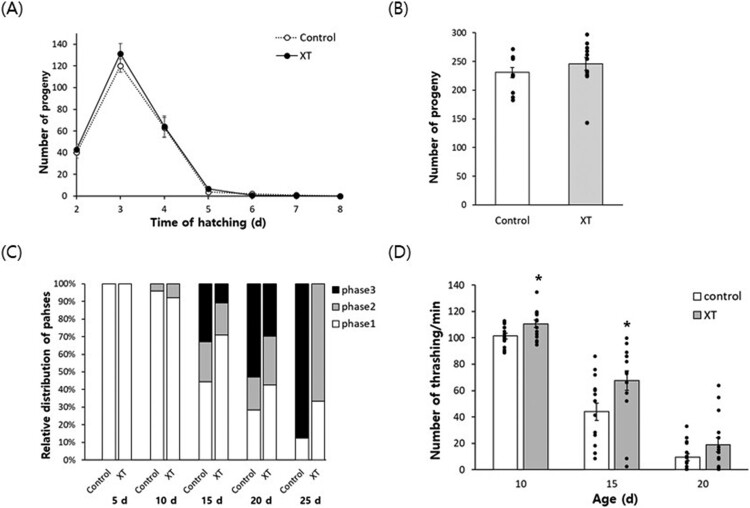
Effect of xanthoxyline on fertility and age-related decline of motility. (A) Number of progeny produced daily (A) and total number of progeny produced throughout a gravid period (B) was compared between the untreated control and xanthoxyline-treated groups. (C) Locomotive activity of worms was classified as phase 1 (spontaneous movement without stimuli), phase 2 (whole body movement with a stimulus), and phase 3 (head-only movement with a stimulus). (D) Numbers of thrashing movements were monitored at 10, 15, and 20 d after hatching. Error bars indicate standard errors. XT, 100 μg/mL xanthoxyline; *, statistically significant compared to the control (*P* < 0.05).

We compared survival after oxidative stress between the untreated control and xanthoxyline-treated groups (Figure 1A). The survival rate of the untreated control was 59.5 ± 3.88% (mean ± standard error). Among the three concentrations of xanthoxyline, only 100 μg/mL xanthoxyline significantly increased the survival rate after oxidative stress to 81.4 ± 6.49% (*P* = 0.044). Xanthoxyline did not increase the survival rate after heat shock and UV irradiation (Figure 1B and C). Indeed, survival after heat shock was decreased by pre-treatment with 10 μg/mL xanthoxyline. Repeated experiments also showed on effect of xanthoxyline on resistance to heat shock or UV irradiation (data not shown). Regarding the effect of xanthoxyline on lifespan, the mean lifespan was increased from 13.8 d in the untreated control to 15.2 d in the xanthoxyline (100 μg/mL)-treated group (*P* < 0.001) (Figure 1D). Independent replications confirmed that xanthoxyline increased the lifespan (Supplementary Table 2). Several lifespan-extending interventions decrease reproduction as a trade-off for longevity (Gruber et al. 2007; Hughes et al. 2007). However, the xanthoxyline-induced longevity phenotype was not accompanied by a reduction in fertility. The daily distribution of progeny number during the gravid period was similar in the xanthoxyline-treated and untreated control (Figure 2A). The total number of progeny produced throughout the gravid period was not affected by xanthoxyline (230.7 ± 9.11 in the untreated control vs. 245.9 ± 11.59 in the xanthoxyline-treated group; *P* = 0.320) (Figure 2B).

### 3.2. Age-related decline of motility was delayed by xanthoxyline

For qualitative analysis of the effect of xanthoxyline on muscle aging, animal locomotive activity was classified into three phases according to the response to mechanical stimuli (Figure 2C). At young ages, 5 and 10 d after hatching, most worms were classified as phase 1 (worms moving spontaneously without stimuli) in both the untreated control and xanthoxyline-treated groups. As worms grew old, the percentage classified as phase 1 decreased, whereas those in phase 2 (whole body movement in the presence of stimuli) and phase 3 (head-only movement in response to stimuli) increased. The age-related decline of motility in the untreated control was delayed by xanthoxyline. For 15-d-old worms, 44.3 and 70.9% of worms were classified as phase 1 in the untreated control and xanthoxyline-treated groups, respectively. The relative percentage of phase 3 was rather decreased from 32.9% in the untreated control to 10.9% in the xanthoxyline-treated group. We also observed a similar pattern, with more phase 1 worms and fewer phase 3 worms in the xanthoxyline-treated group, for 20-d-old animals. Interestingly, there were no phase 2 worms, and 87.5% of worms were classified as phase 3 in the 25-d-old untreated control, while there were no phase 3 worms and 66.7% of phase 2 worms in xanthoxyline-treated worms at the same age. For a quantitative analysis, the number of thrashing movements was counted at different ages. As shown in Figure 2D, number of thrashing movements was reduced as animals aged in both the untreated control and xanthoxyline-treated group. However, the comparison of thrashing movements at the same age revealed that xanthoxyline can confer a positive effect on motility. For 10-d-old animals, the number of thrashing movements increased from 101.4 ± 2.10–110.6 ± 2.84 by xanthoxyline supplementation (*P* = 0.015). A significant increase in the number of thrashing movements was also observed in 15-d-old animals: 44.1 ± 6.55 in the untreated control vs. 67.5 ± 7.40 in the xanthoxyline-treated group (*P* = 0.025). The number of thrashes by 20-d-old worms was non-significantly increased by xanthoxyline from 9.5 ± 2.72–18.7 ± 5.38 (*P* = 0.138) (Figure 2D).

### 3.3. Xanthoxyline induced the expression of longevity-assuring genes

**Figure 3.**
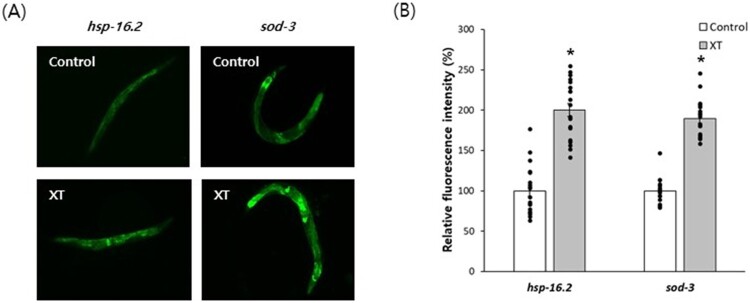
Increased expression of longevity-assuring genes by xanthoxyline. (A) GFP under the control of the *hsp-16.2* or *sod-3* promoter was observed in 7-d-old worms by fluorescence microscopy. (B) Relative fluorescence intensities of the untreated control and xanthoxyline-treated groups were compared. Error bars indicate standard errors. XT, 100 μg/mL xanthoxyline; *, statistically significant compared to the control (*P* < 0.05).

Genetic screening for longevity-assuring genes identified two genes, *hsp-16.2* and *sod-3*, whose expression levels are positively correlated with the remaining lifespan (Rea et al. 2005; Sanchez-Blanco & Kim 2011). We examined the effect of xanthoxyline on the expression of *hsp-16.2* and *sod-3*. The expression of GFP induced by *hsp-16.2* or *sod-3* promoter was upregulated by xanthoxyline (Figure 3A). Under the control of the *hsp-16.2* promoter, the relative expression of GFP in the xanthoxyline-treated group was 200.3 ± 7.90 compared to the untreated control (100.0 ± 6.72, *P* < 0.001). Xanthoxyline increased GFP expression 1.9-fold with the *sod-3* promoter (100.0 ± 3.46 in the untreated control vs. 189.4 ± 5.33 in the xanthoxyline-treated group; *P* < 0.001) (Figure 3B).

### 3.4. The xanthoxyline-mediated lifespan extension is dependent on BEC-1 and DAF-16

**Figure 4.**
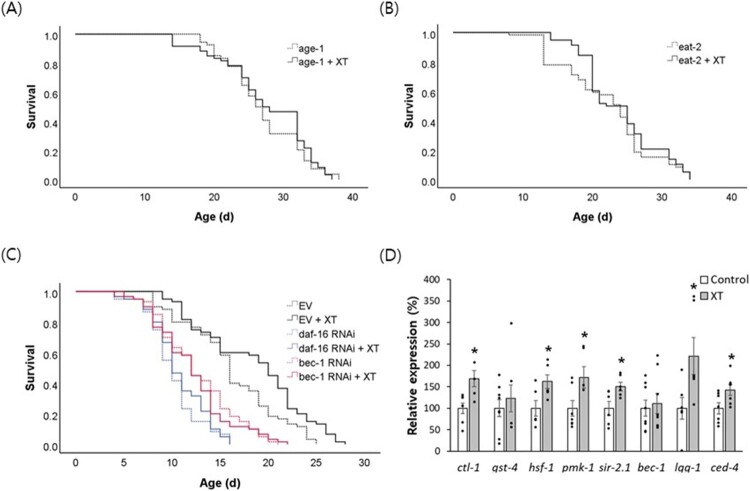
Genetic analysis of lifespan extension by xanthoxyline. Effect of xanthoxyline on the lifespan of *age-1* (A) and *eat-2* (B) mutants were examined. (C) Survival of worms fed empty vector, *bec-1* RNAi clone, or *daf-16* RNAi clone was compared between the untreated control and xanthoxyline-treated groups. (D) Quantitative RT-PCR was performed to examine the effect of xanthoxyline on gene expression. Relative expression levels in worms treated with xanthoxyline were calculated with those in the untreated control set to 100. Error bars indicate standard errors. Kaplan-Meier analysis was used for statistical analysis of survival curve. XT, 100 μg/mL xanthoxyline; EV, empty vector.

To identify the mechanisms underlying the xanthoxyline-induced lifespan extension, we assayed its effect on the lifespan of long-lived mutants. The lifespan of *age-1* (*hx546*), which prolongs lifespan by reducing insulin/IGF-1-like signaling, was unaffected by xanthoxyline (*P* = 0.504) (Figure 4A and Supplementary Tabel 3) (Johnson 1990). Xanthoxyline also failed to increase the lifespan of *eat-2* (*ad465*), a genetic model of DR (*P* = 0.261) (Figure 4B and Supplementary Table 3) (Uno & Nishida 2016). These findings suggest that the lifespan-extending effect of xanthoxyline is mediated by common pathways in the two genetic mutants tested. Interestingly, genetic knockdown of *bec-1*, an autophagy gene, abolished the longevity phenotype conferred by xanthoxyline (Figure 4C). The mean lifespan of worms fed a *bec-1* RNAi clone (12.5 d) was not significantly different from that of worms fed a *bec-1* RNAi clone and xanthoxyline simultaneously (12.1 d, *P* = 0.664). In worms in which *daf-16* (a FOXO transcription factor that modulates stress responses) was suppressed, xanthoxyline did not prolong the lifespan (mean 10.1 and 10.7 d in the untreated control and xanthoxyline-treated groups, respectively; *P* = 0.352) (Figure 4C). The results of repeated experiments are shown in the supplementary data (Supplementary Table 4).

### 3.5. Xanthoxyline induced the expression of age-related genes and activated autophagy

**Figure 5.**
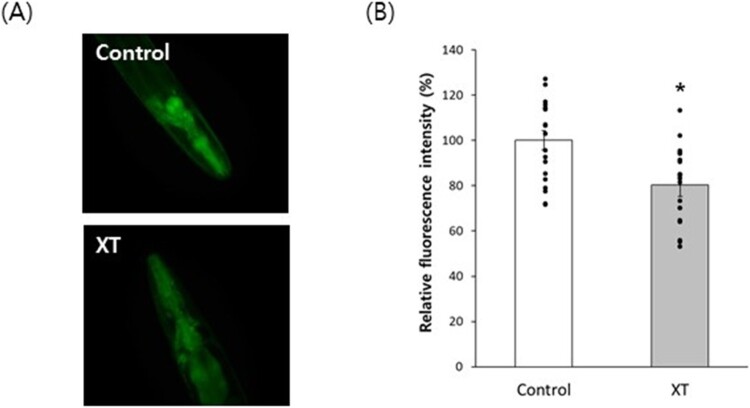
Activation of autophagy by xanthoxyline. (A) Representative images of BC12921 in the untreated control and xanthoxyline-treated groups. (B) Relative fluorescence intensities. Error bars indicate standard errors. XT, 100 μg/mL xanthoxyline; *, statistically significant compared to the control (*P* < 0.05).

Quantitative RT–PCR showed that xanthoxyline increased the expression of the stress-responsive genes, *ctl-1* (1.7-fold, *P* = 0.009) and *hsf-1* (1.6-fold, *P* = 0.026) (Figure 4D). PMK-1, an upstream activator of SKN-1 that regulates oxidative stress and DR responses, was 1.7-fold up-regulated by xanthoxyline (*P* = 0.035). The expression of another mediator of the DR response, SIR-2.1, was induced 1.5-fold by xanthoxyline (*P* = 0.023). The expression of *lgg-1* involved in autophagosome formation was increased up to 2.2-fold compared to the untreated control (*P* = 0.035). Interestingly, xanthoxyline induced by 1.4-fold the expression of *ced-1*, an apoptotic gene induced by elevation of mitochondrial ROS (*P* = 0.038). (Figure 4D). We investigated the effect of xanthoxyline on autophagy using the BC12921 strain that expresses fluorescent SQST-1, a substrate protein for autophagy. Xanthoxyline decreased the expression of SQST-1 (Figure 5A). Compared to the untreated control (100.0 ± 4.19), the xanthoxyline-treated group showed a significantly reduced fluorescence intensity (80.2 ± 4.95, *P* = 0.002) (Figure 5B).

### 3.6. The degeneration of dopaminergic neurons was blocked by xanthoxyline

**Figure 6.**
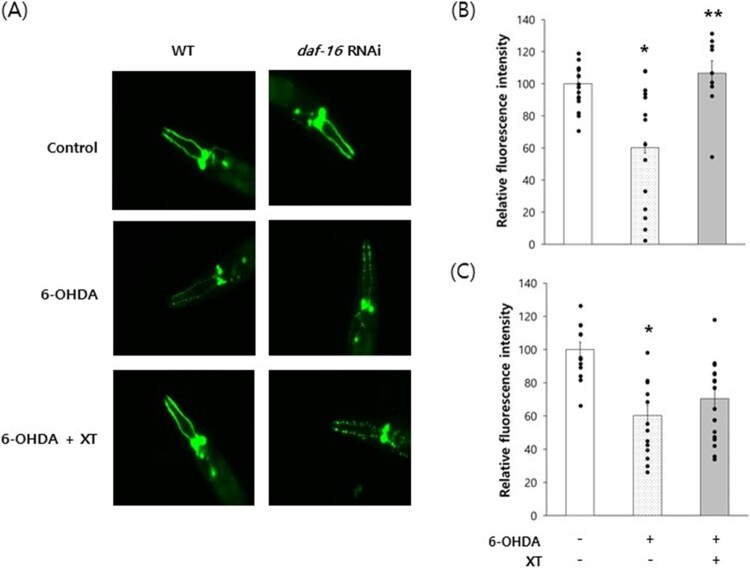
DAF-16-dependent inhibition of dopaminergic neurodegeneration by xanthoxyline. (A) Dopaminergic-specific neurodegeneration was induced by 6-OHDA. The experiments were repeated using *daf-16*-knockdown worms. Fluorescence was quantified using Image-J software (B & C). Error bars indicate standard errors. XT, 100 μg/mL xanthoxyline; 6-OHDA, 6-hydroxydopamine; *, statistically significant compared to the control (*P* < 0.05); **, statistically significant compared to the 6-OHDA-treated group (*P* < 0.05).

Dopaminergic-neuron-specific degeneration in PD was prevented by xanthoxyline. Treatment of wild-type N2 with 6-OHDA led to degeneration of dopaminergic neurons, an effect inhibited by simultaneous treatment with xanthoxyline (Figure 6A). The relative GFP fluorescence intensity in dopaminergic neurons was 100 ± 9.76 in the untreated control and 60.3 ± 3.51 in the 6-OHDA-treated group (*P* = 0.003). The reduction in fluorescence intensity caused by 6-OHDA was restored by xanthoxyline to 106.4 ± 7.95 (*P* = 0.006 compared to the 6-OHDA-treated group) (Figure 6B). DAF-16 is required for neuroprotective effects of gastrodin, a plant-derived antioxidant, in models of PD (Yan et al. 2019). In this study, the prevention by xanthoxyline of dopaminergic neurodegeneration was abolished by the repression of *daf-16* expression (Figure 6A). In worms fed a *daf-16* RNAi clone, the reduction in fluorescence intensity from 100.0 ± 4.36–60.4 ± 6.04 by 6-OHDA (*P* < 0.001) was not restored by xanthoxyline (70.4 ± 5.38, *P* = 0.225 compared to the 6-OHDA-treated group) (Figure 6C).

## 4. Discussion

The free radical theory suggests that accumulation of oxidative damage caused by ROS is a pivotal cause of aging and that modulation of cellular antioxidant defense could regulate aging and prolong the lifespan. Phytochemicals are plant bioactive compounds, many of which are antioxidants. The health benefits of polyphenols, flavonoids, and carotenoids have been investigated, including their effects on lifespan. Resveratrol and myricetin, polyphenolic phytochemicals, exerted antioxidant and lifespan-extending effects in *C. elegans* (Buchter et al. 2013; Zhou et al. 2021). The plant-derived flavonoids, catechin, quercetin, and kaempferol, increased both resistance to oxidative stress and lifespan (Pallauf et al. 2017). In this study, xanthoxyline increased resistance to oxidative stress *in vivo* and extended the lifespan. Xanthoxyline is a carboxylic ester, and the effects of phytochemicals are dependent on their chemical structure, so a structure–activity relationship study of xanthoxyline is needed. Many lifespan-extending genetic or nutritional interventions also reduce reproduction as a trade-off for longevity. The *age-1* mutant exhibited an increased lifespan due to decreased insulin/IGF-1-like signaling and produced fewer progeny (Hughes et al. 2007). Dietary supplementation with resveratrol, fisetin, or butein conferred a longevity phenotype, which was paid for by deceased fertility (Park et al. 2022; Kim et al. 2024). However, the lifespan-extending effect of xanthoxyline was not accompanied by a reduction in reproduction. Blueberry polyphenols delay the decline of pharyngeal pumping as a trade-off for longevity (Wilson et al. 2006). The lifespan extension by xanthoxyline might have a trade-off other than by decreased fertility. Age-related alterations in muscle include atrophy and reduced strength, which are related to ROS accumulation and used as biomarkers of aging (Del Campo et al. 2016). Animals with a knockout of Cu/Zn SOD showed premature muscular dysfunction as a result of increased levels of ROS. By contrast, supplementation with antioxidant phytochemicals, such as fisetin and phlorizin, inhibited the degeneration of muscle function (Park et al. 2022; Park & Park 2022). Xanthoxyline also delayed the age-related decline of motility. Therefore, xanthoxyline exerts an anti-aging effect in *C. elegans*, possibly via its antioxidant activity. Further studies with other biomarkers of aging and model organisms are needed to confirm the anti-aging effect of xanthoxyline.

Several lifespan-extending effects, including reduced insulin/IGF-1-like signaling and DR, are conserved in several model organisms (Riera et al. 2016). In *C. elegans*, mutations in *daf-2* or *age-1*, a plasma-membrane receptor and an intracellular mediator of insulin/IGF-1-like signaling, respectively, led to nuclear translocation of DAF-16, a FOXO transcription factor that regulated expression of multiple stress-responsive genes (Johnson et al. 2001). The DR-induced longevity phenotype is mediated by *sir-2.1*, a key metabolic sensor (Wood et al. 2004). Our genetic analysis revealed that the xanthoxyline-induced lifespan extension overlapped with reduced insulin/IGF-1-like signaling and DR. Genetic knockdown of *daf-16* abolished the longevity phenotype conferred by xanthoxyline and induced the expression of downstream targets of DAF-16 (*ctl-1*, *sod-3*, and *hsp-16.2*). The expression of two genes involved in DR responses, *sir-2.1* and *pmk-1*, was significantly increased by xanthoxyline. Activation of autophagy is involved in lifespan extension by reduced insulin/IGF-1-like signaling and DR (Toth et al. 2008). The lifespan extension caused by *daf-2* mutation was inhibited by repression of *bec-1*, *atg-8*, or *lgg-1* (Toth et al. 2008). Similarly, the prolonged lifespan of an *eat-2* mutant was abolished by genetic knockdown of *bec-1* (Toth et al. 2008). The abolition by *bec-1* RNAi of the xanthoxyline-mediated lifespan extension and the induction of *lgg-1* expression suggest that autophagy is involved in the longevity phenotype mediated by xanthoxyline. The reduced protein level of SQST-1, an autophagy substrate, in long-lived xanthoxyline-treated animals supports the hypothesis that activation of autophagy is required for the xanthoxyline-mediated extension of lifespan.

PD is the second most common age-related neurodegenerative disease for which there are no effective and safe therapeutics. Because of the side effects of current treatments for PD, including dopamine and levodopa, research has focused on identifying a novel therapeutic compound from natural sources. Gastrodin, an antioxidant and anti-inflammatory compound in the orchid *Gastrodia elata Blume*, inhibited the accumulation of α-synuclein and degeneration of dopaminergic neurons via insulin/IGF-1-like signaling (Yan et al. 2019). Extracts from Ayurvedic herbs exerted neuroprotective effects in a PD model (Anjaneyulu et al. 2020). Cannabidivarin, a neuroprotective analog of cannabidiol found in *Cannabis sativa*, prevented oxidative stress and recovered dopaminergic neuronal damage in a manner involving DAF-16 (Wang et al. 2022). In this study, xanthoxyline reversed the degeneration of dopaminergic neurons caused by 6-OHDA. Genetic knockdown of *daf-16* abolished the effect of xanthoxyline in a PD model. Taken together, our data suggest that xanthoxyline protects dopaminergic neurons in a manner dependent on DAF-16. The neuroprotective effect of cannabidiol was accompanied by restoration of the locomotion rate in *C. elegans* (da Cruz Guedes et al. 2023). Also, supplementation with gastrodin prevented the decreased chemotaxis in PD (Yan et al. 2019). In this study, xanthoxyline prevented the age-related decline of locomotive and thrashing activities. Therefore, the locomotion rate has potential for use in screening of candidate compounds against PD.

Although our results suggest that xanthoxyline positively affects aging and lifespan, direct causation in humans is challenging to establish conclusively because of the complexity of aging and lifespan determination. Moreover, the effects of xanthoxyline can vary depending on the amount consumed, genetic factors, lifestyle factors, and overall diet quality. In summary, xanthoxyline has potential for promoting health and extending lifespan; long-term human studies are needed to investigate further its properties.

## Supplementary Material

Supplementary Material
