# Supplementary material for: Xanthoxyline prevents aging and neuronal damage by activating autophagy and DAF-16 expression in Caenorhabditis elegans
Source: Anim Cells Syst (Seoul). 2025 Aug 22;29(1):544–55. doi: 10.1080/19768354.2025.2549756 (PMC12377130; doi:10.1080/19768354.2025.2549756)
Supplement: Supplementary Material [file TACS_A_2549756_SM7792.docx]

**Supplementary Table 1.** Primer set of each gene used for quantitative RT-PCR

| **Gene Name** | **Primer** | **Sequence** |
| --- | --- | --- |
| *ama-1* | forward | 5'-CGG AGC AGC CAG GAA CTT C-3' |
|  | reverse | 5'-AAC GGG AAA AAT CTT ATG AAT-3' |
| *ctl-1* | forward | 5'-AAT GGA TAC GGA GCG CAT AC-3' |
|  | reverse | 5'-GCG TCA GTT GGA TCG AGA TT-3' |
| *gst-4* | forward | 5'-GCT GAA GCC AAC GAC TCC AT-3' |
|  | reverse | 5'-GAC CGA ATT GTT CTC CAT CGA-3' |
| *hsf-1* | forward | 5'-ATG CAG CCA GGA TTG TCG AA-3' |
|  | reverse | 5'-GCA CGT TTT GAG TTG GGT CC-3' |
| *pmk-1* | forward | 5'-CGA CTC GAC GAG AAG GAT-3' |
|  | reverse | 5'-ATA TGT ACG ACG GGC ATG-3' |
| *sir-2.1* | forward | 5'-TCC AAG GCT GCT GAG CAT TAC TA-3' |
|  | reverse | 5'-TGA TGA GCA AGA CGA ACC ACA CA-3' |
| *bec-1* | forward | 5'-AGG AGC TGG AGC AAC AGT TGA AGA-3' |
|  | reverse | 5'-ATA TTG ACG TTC GGC TTC CAG CGA-3' |
| *lgg-1* | forward | 5'- AAC AAC TTT GAG AAG CGT CGT GCC -3' |
|  | reverse | 5'- TCT TCT GGA CGA AGT TGG ATG CGT -3' |
| *ced-4* | forward | 5'-AGT GCT CTT GCT TTC GCA GTT GTG-3' |
|  | reverse | 5'-TGA GAA GAG CTC CAC GTT TGC TGA-3' |

**Supplementary Table 2.** Effect of xanthoxyline on lifespan in *C. elegans*

|  | **Xanthoxyline (μg/mL)** | **Mean lifespan (d)** | ***P* value^1)^** | **% effect^2)^** |
| --- | --- | --- | --- | --- |
| 1^st^ experiment | 0 | 17.6 |  |  |
|  | 100 | 19.0 | 0.006 | 7.9 |
| 2^nd^ experiment | 0 | 13.8 |  |  |
|  | 100 | 15.2 | < 0.001 | 9.6 |

^1)^ *P* value was calculated using Kaplan-Meier analysis by comparing the survival of the untreated control group (0 μg/mL xanthoxyline) to that of xanthoxyline-treated group (100 μg/mL xanthoxyline).

^2)^ % effects were calculated by (*C*-*X*)/*C**100, where *X* is the mean lifespan of xanthoxyline-treated group and *C* is the mean lifespan of the untreated control group.

**Supplementary Table 3.** Effect of xanthoxyline on lifespan in long-lived mutants

|  |  | **Mean lifespan (d)** | |  |
| --- | --- | --- | --- | --- |
|  | **Mutants** | **Control** | **Xanthoxyline (100 μg/mL)** | ***P* value^1)^** |
| 1^st^ experiment | WT | 20.5 | 21.8 | 0.003 |
|  | *age-1* | 27.1 | 27.6 | 0.504 |
|  | *eat-2* | 22.1 | 23.9 | 0.261 |
| 2^nd^ experiment | WT | 18.3 | 19.3 | 0.021 |
|  | *age-1* | 31.1 | 28.3 | 0.080 |
|  | *eat-2* | 22.4 | 22.6 | 0.167 |

^1)^ *P* value was calculated using Kaplan-Meier analysis by comparing the survival of the untreated control group (0 μg/mL xanthoxyline) to that of xanthoxyline-treated group (100 μg/mL xanthoxyline).

**Supplementary Table 4.** Effect of *daf-16* or *bec-1* knockdown on lifespan extension by xanthoxyline

|  |  | **Mean lifespan (d)** | |  |
| --- | --- | --- | --- | --- |
|  | **RNAi** | **Control** | **Xanthoxyline (100 μg/mL)** | ***P* value^1)^** |
| 1^st^ experiment | EV | 16.1 | 18.4 | 0.005 |
|  | *daf-16* | 10.1 | 10.7 | 0.352 |
|  | *bec-1* | 12.5 | 12.1 | 0.664 |
| 2^nd^ experiment | EV | 20.5 | 23.4 | < 0.001 |
|  | *daf-16* | 10.6 | 10.9 | 0.655 |
|  | *bec-1* | 22.3 | 20.8 | 0.137 |

^1)^ *P* value was calculated using Kaplan-Meier analysis by comparing the survival of the untreated control group (0 μg/mL xanthoxyline) to that of xanthoxyline-treated group (100 μg/mL xanthoxyline).
